# Phase-change devices for simultaneous optical-electrical applications

**DOI:** 10.1038/s41598-017-10425-8

**Published:** 2017-08-29

**Authors:** Yat-Yin Au, Harish Bhaskaran, C. David Wright

**Affiliations:** 1 Department of Engineering, University of Exeter, Exeter, EX4 4QF UK; 2 Department of Materials, University of Oxford, Oxford, OX1 3PH UK

## Abstract

We present a viable pathway to the design and characterization of phase-change devices operating in a mixed-mode optical-electrical, or optoelectronic, manner. Such devices have potential applications ranging from novel displays to optically-gated switches to reconfigurable metamaterials-based devices. With this in mind, a purpose-built optoelectronics probe station capable of simultaneous optical-electrical excitation and simultaneous optical-electrical response measurement has been designed and constructed. Two prototype phase-change devices that might exploit simultaneous optical and electrical effects and/or require simultaneous optical and electrical characterisation, namely a mixed-mode cross-bar type structure and a microheater-based structure, have been designed, fabricated and characterized. The microheater-based approach was shown to be capable of successful thermally-induced cycling, between amorphous and crystalline states, of large-area phase-change devices, making it attractive for practicable pixel fabrication in phase-change display applications.

## Introduction

Phase-change devices are of much current interest in a number of important application areas including non-volatile memories^1–3^, optoelectronic displays^4^ and so-called phase-change-based metadevices^5–7^. In non-volatile memory applications phase-change devices are switched between two stable resistance states and were initially seen as a possible replacement for, or complement to, conventional silicon CMOS ‘flash’ memories, but their potential as a competitor for silicon DRAM (dynamic random access memory) has also been investigated^8^. More recently, phase-change devices have also emerged as one of the favourites for a new entry into the established memory technology hierarchy, namely the so-called ‘storage class memory’ that offers performance attributes lying somewhere between those of DRAM and those of magnetic hard disk storage^9^. In non-volatile memory applications it is the electrical switching of a phase-change cell between its high-resistance amorphous (or RESET) state and its low-resistance crystalline (or SET) state that forms the basis of the memory process. Other application areas, however, are more concerned with the change in optical properties brought about by a change of phase; indeed, this forms the basis of the operation of several re-writable optical disk formats that have been with us for many years, e.g. DVD-RW and Blu-ray RW. More recently, the resistive-switching of phase-change cells incorporated into a type of Fabry-Perot resonator structure was shown to provide the capability for new types high-resolution, fast and non-volatile optoelectronic colour displays and holograms^4, 10^. Another interesting recent development that also exploits the large change in optical properties resulting from a switch between amorphous and crystal states is that of phase-change metadevices. Here, phase-change materials are typically used as a form of switchable dielectric that, in combination with optical metasurfaces, can provide a wide range of novel functionalities such as beam steering with no moving parts^11^, ‘perfect’ infrared absorbers and modulators^7, 12^ and planar and re-configurable thin-film lenses^6, 13^.

Thus, it is clear that the ability of phase-change materials to (i) provide simultaneous changes in both optical (refractive index) and electrical (resistivity) properties when switched between amorphous and crystalline states, (ii) be repeatedly and reversibly switched between those states and (iii) exist stably in, and be relatively easily excited into, intermediate or fractionally-crystallized states, leads to a host of new application possibilities in the area of optoelectronic devices and systems. It is therefore important to be able to design and fabricate phase-change devices capable of functioning simultaneously under both optical and electrical excitations. It is also important to be able to characterize and understand their simultaneous optical and electrical response. In this paper, therefore, we report on (i) the details of a combined optical and electrical test station purpose-built for use in the evaluation and characterization of mixed-mode optical-electrical phase-change devices, (ii) the electrical response of optically-switched nanoscale phase-change cross-bar devices (with possible applications in the area of optically-gated switches) and (iii) the optical response of thermo-electrically switched microscale phase-change devices (with possible phase-change display applications).

The study of optically-induced changes in the electrical properties of phase-change devices is appealing in a number of respects. In particular, while conventional phase-change devices such as memories are invariably two-terminal devices, the introduction of a third terminal (or gate) to the device could provide interesting new possibilities, such as ultra-fast optically-triggered phase-change switches, 3-terminal phase-change logic and even a form of chalcogenide-based field effect transistor^14^. Indeed, 3-terminal phase-change devices have already been explored in the area of RF-switching, where the 3^rd^ terminal is essentially used as an electrical heater to thermally switch a phase-change cell between high and low resistance states, so providing high or low insertion losses for RF signals applied to the device^15–17^. In such thermally-switched 3-terminal devices, however, the switching times for the phase-change cell are relatively long, typically microseconds. Optically-triggered switches could potentially deliver much faster switching times, since it is known that using ultra-fast laser pulses the switching time for phase-change materials can be brought down to the picosecond regime^18, 19^. On the other hand, thermo-electrically switched phase-change devices could be particularly useful in phase-change display applications. This is because for the efficient and effective pixel coverage of many common display screens, pixel sizes are in the (many) micrometre range; e.g. 4 K UHD screens have approximately 10^7^ to 10^9^ pixels per square metre (or pixel areas of at least 1000 µm^2^). But, the direct electrical switching of large-area phase-change devices, particularly in the cross-bar configuration (as used to date in display applications^4, 20^) and using Ge_2_Sb_2_Te_5_ alloys is complicated by the filamentary nature of crystallization and the difficulty in obtaining uniform temperature distributions throughout the entire volume of the phase-change material (such that while some regions of the device may at a particular time instant be at a suitable temperature for say amorphization, other regions will at the same time be at temperatures conducive to crystallization). Indirect thermo-electric switching of phase-change devices can potentially avoid such problems, leading to the successful realization of large area phase-change pixels, as we later show.

In addition to such interesting application areas for simultaneous electrical and optical operation of phase-change devices, the ability to access both excitation and detection modes at the same time should prove useful in clarifying some unresolved aspects of the switching process in phase-change devices, such as the role of heat and the electric field on the so-called threshold-switching mechanism^21^.

## Results and Discussion

### Combined optical and electrical test station

The characterisation and testing of mixed-mode phase-change devices requires a test platform that allows for simultaneous electrical and optical access. Combined optical-electrical test systems have of course been described before for various non phase-change device studies (e.g. Yuan *et al*.^22^ examined infrared photogalvanic current in WSe_2_ transistors, Vicarelli *et al*.^23^ observed terahertz-light-induced electrical signals in graphene-based transistors). In addition, a very recent publication reported on a mixed-mode study of phase-change cross bar devices^24^. However, the system we describe here is significantly different to such previous studies. In particular, our system includes the important facility for dynamic/time-resolved measurements on the nanosecond (or even subnanosecond) timescale (not included in the work of ref. 24) which is most important for understanding device switching dynamics. Moreover, we used a free-space optics approach combined with a high numerical aperture objective lens and short wavelength (405 nm) laser excitation. This not only provides a significantly superior optical resolution *cf*. previous work, so enabling the investigation of state-of-the-art phase-change devices (that typically have sizes measured in the hundreds of nanometre range), but also provides for a more flexible system with increased functionality (e.g. a simultaneous white light imaging capability, the ability to extract spectral information from the optical signal reflected from the device, the ability to test a very wide range of device sizes and types).

**Figure 1:**
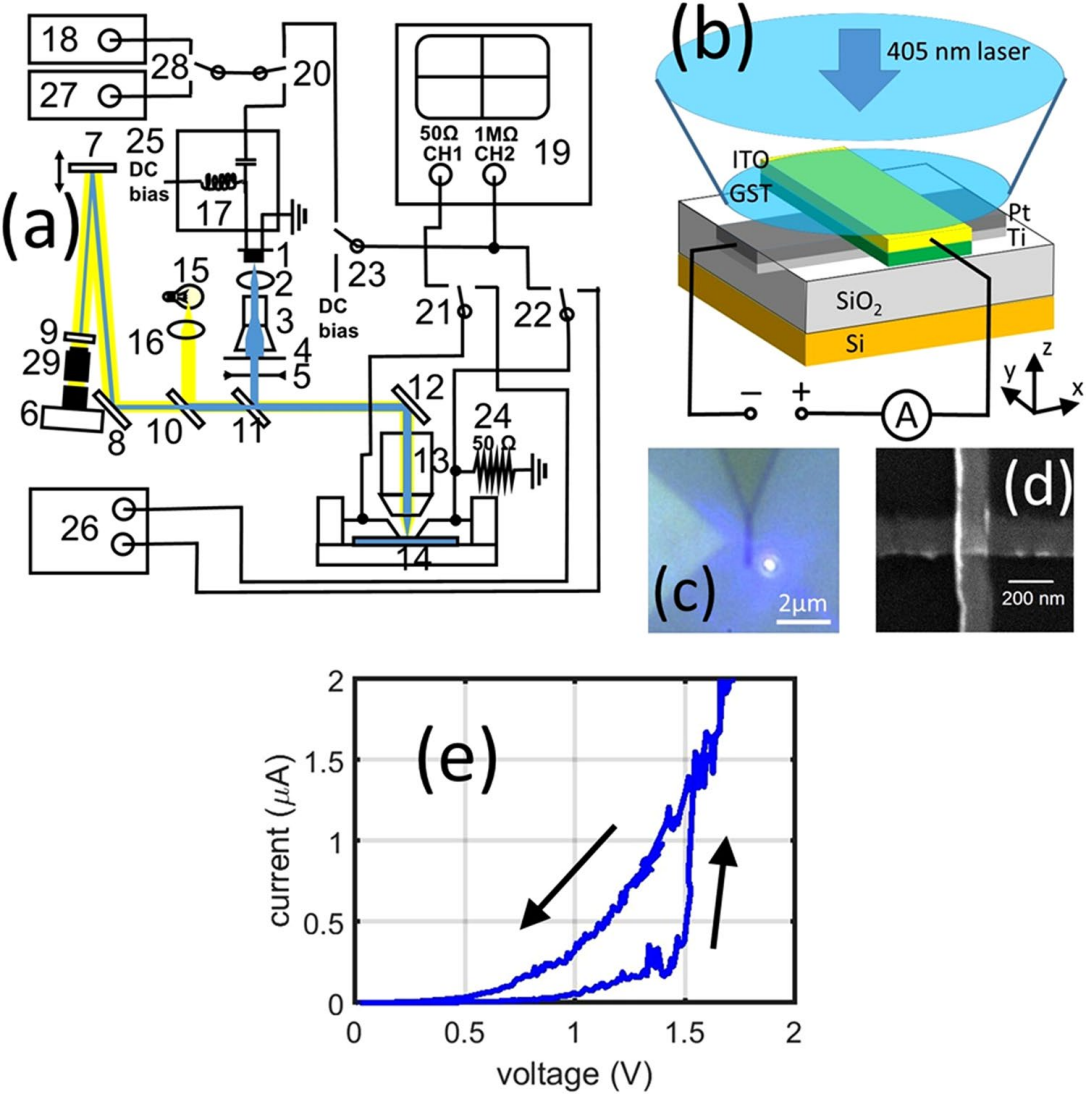
The purpose-built combined electrical-optical test system. (**a**) Schematic of the overall system showing (1) Laser diode, (2) converging lens, (3) beam expander, (4) pin hole, (5) shutter, (6) CMOS camera, (7) and (8) mirror, (9) 450 nm long pass filter, (10) beam splitter, (11) 425 nm long pass beam splitter, (12) mirror, (13) objective, (14) sample, (15) white light source, (16) converging lens, (17) bias tee, (18) Avtech pulse generator, (19) oscilloscope, (20) to (23) rf relays, (24) 50 Ω resistor, (25) dc bias source for laser diode, (26) Keithley source meter (27) Tektronix function generator (28) rf relay, and (29) eye piece. (**b**) Illustration of a typical simultaneous electrical-optical experiment, here showing a specially-designed phase-change cross-bar device under optical excitation by the 405 nm laser while the device resistance is monitored in real-time. (**c**) Optical image of the cross bar structure accompanied by the laser spot. (**d**) SEM image of an exemplar cross bar device, here with lateral dimension slightly less than 200 nm. (**e**) A typical I-V curve of a cross-bar device obtained using standard electrical-only excitation.

Our test station is shown in block diagram form in Fig. 1. With respect to optical access, an expanded and collimated 405 nm wavelength laser beam is reflected by a mirror onto a high numerical aperture microscope objective lens and focused to a diffraction-limited spot on the sample/device. Precise focusing of the laser is achieved by moving the objective vertically (z-direction) using a combination manual and piezo stages. Similarly, precise positioning of the sample/device is achieved using a combination of horizontal (x-y) manual and piezo stages. Electrical access to devices is provided via two RF probes of the ground-signal-ground (GSG) type. The probes are mounted on two xyz manual micro-stages to provide precise control of the probe position in order to align and touchdown with the device. The devices themselves are mounted on a coplanar waveguide (CPW) whose conductors match the pitch of the GSG probe connections. The laser diode is mounted on a bias Tee and is biased at sub-threshold state (to allow for fast switching) by a constant current source. Electrical SET and RESET pulses are provided by two separate pulse generators connected to the same bias Tee via an RF relay. A computer controlled shutter is installed in the laser beam pathway and is opened only when the pulse generators are triggered (so as to avoid any undesired spurious laser irradiation reaching the sample/device). A high resolution/high speed CMOS camera and a white light source also allows the device being tested to be imaged in real time. Figure 1(b) illustrates the basic concept of the simultaneous optical-electrical investigations at the heart of the paper, here for the case of a phase-change cross-bar device (see Fig. 1(c) and (d)) undergoing optical excitation while its electrical properties are concurrently investigated.

The maximum laser power that can be delivered to the sample (i.e. the power after the objective lens) in our purpose-built test system is around 60 mW (more than enough to reach the GST melting temperature) and the optical excitation can vary from continuous wave down to pulses in the sub-nanosecond region, thus giving us access to an exceptionally wide range of optical excitation conditions. Electrical excitation is possible using voltages in the range −10 to 20 V and pulse durations again down to the sub-nanosecond region (electrical rise/fall times of 0.2 ns). Indeed, the ‘conventional’ electrical testing of devices is straightforward in our system and standard electrical measurements often carried out on resistive-switching memory devices (such as I-V curves, SET/RESET cycling etc.) are a useful way of providing an initial screen of devices to check that they are functioning correctly. For example, in Fig. 1(e) we show an I-V curve for a phase-change cross-bar device (having the structure illustrated in Fig. 1(b)); it displays the characteristic threshold switching effect expected of electrical phase-change devices^2, 8^.

### Mixed-mode cross-bar devices and the optically-gated switch

To allow for both simultaneous optical and electrical access in a cross-bar device configuration we designed and fabricated devices having optically-transparent, electrically-conductive ITO (indium tin oxide) top electrodes, as seen in Fig. 1(b). A typical cross-bar device structure thus comprised a Si substrate with a 50 nm thermal oxide layer, a Pt bottom electrode (typically of 5 nm thickness and with a 5 nm Ti adhesion layer), a Ge_2_Sb_2_Te_5_ (GST) phase-change layer (typically of 15 nm thickness) and an ITO top electrode (typically around 30 nm in thickness). The Ti/Pt bottom electrode is deposited into a 10 nm deep trench (etched into the SiO_2_ layer by reactive ion etching), so as to planarize the bottom device surface. The image of a typical device, as captured by the *in-situ* CMOS camera of our mixed-mode test station, is shown in Fig. 1(c). An SEM image of the same device is shown in Fig. 1(d) and reveals the Ti/Pt and GST/ITO arm widths to be 175 nm and 150 nm respectively in this case. Further details of the device fabrication process can be found in the Methods section and the Supplementary Information.

**Figure 2:**
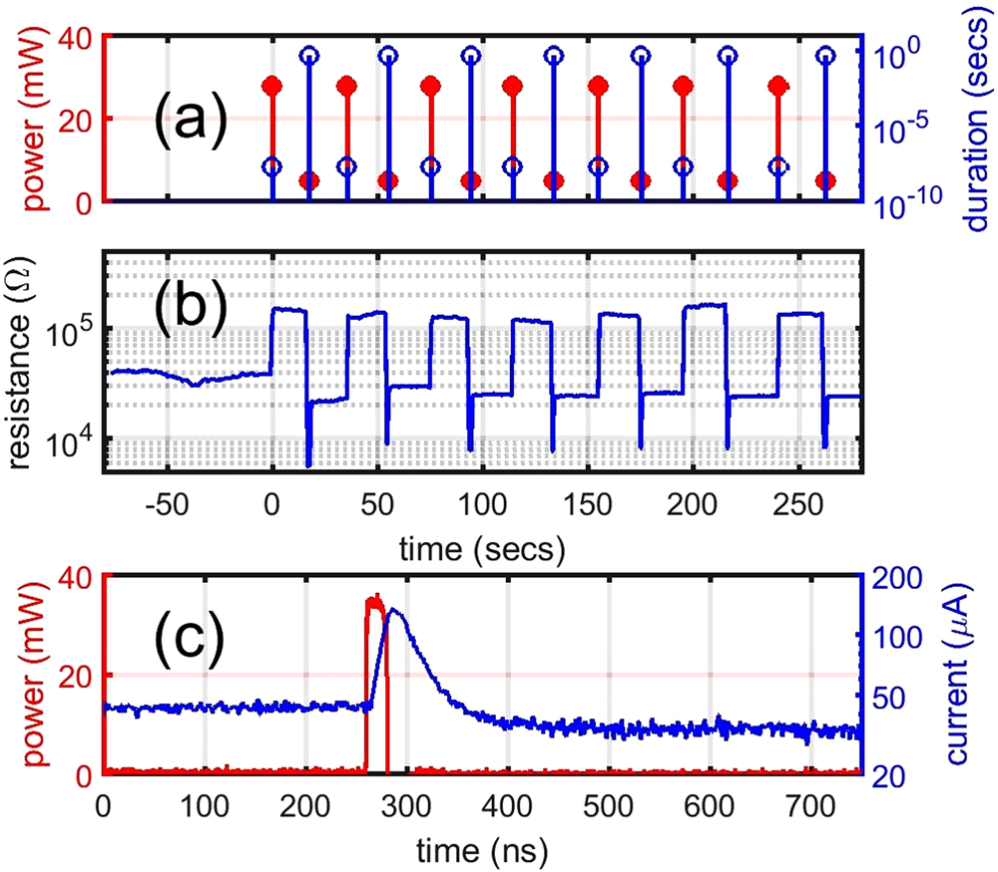
Simultaneous optical-electrical operation of mixed-mode cross-bar devices. The dimension of the device under test is 1 × 1 µm^2^ in size. (**a**) Duration and power of laser pulses applied to the cross-bar device during the mixed-mode switching experiments. (**b**) Real time monitoring of device resistance, showing controllable switching between high and low state when appropriate excitation is being applied. Sensing voltage equal 0.1 V was applied. (**c**) Time resolved measurement of the cross-bar device under a single laser pulse. The device was biased at 0.5 V and the current started out at 43 µA and ended up at 32 µA, indicating an increase of resistance and manifests a partial RESET process.

We now turn our attention to the simultaneous optical-electrical behaviour of our mixed-mode phase-change devices, here considering the case whereby excitation is optical but detection is electrical, i.e. we use optical switching with simultaneous real-time readout of the device resistance. The cross-bar cells were in the amorphous state as fabricated. We then applied long-duration, low power laser pulses to bring the cell into a stable SET state. RESET laser excitations were subsequently applied, starting with relatively low power and then increasing gradually until suitably high resistances were obtained (indicating that significant re-amorphization had occurred). The application of such SET and RESET excitations was then repeated while monitoring the resistance of the cell. In Fig. 2 we show the result of such a measurement applied to an exemplar device (note that we fabricated over 20 similar devices with dimensions from 150 × 150 nm^2^ to 1 × 1 µm^2^ and all exhibited similar behaviour). Here the SET process involved excitation of the cell with a laser pulse of 5 mW and 0.5 secs in duration, while the RESET process involved a single laser pulse of 28 mW and 20 ns duration (see Fig. 2(a)). In Fig. 2(b) it can be seen that the device successfully cycles between high resistance and low resistance states using this optical excitation routine, though in this case the resistance change, at just less than one order of magnitude, is relatively small, indicating that for the RESET conditions used only a partial re-amorphization occurs. We also note that overshooting of resistance drop in the SET process is observed, which we attribute to photocurrent effects under illumination by the SET laser pulse (which lasts for 0.5 secs as mentioned earlier).

The results of Fig. 2(b) demonstrate that suitably designed phase-change cross-bar cells can deliver optically-induced changes in electrical resistance. However, for potential applications of such optically-driven electrical changes (for example the optically-gated switches discussed in the introduction) it is also important to understand and be able to measure the detailed dynamic response of devices so as to determine, for example, the possible switching speeds that might be achievable. We show just such a dynamic response in Fig. 2(c). Here an optical excitation pulse of 20 ns duration at 35 mW is applied to the device while it is electrically biased at 0.5 V so as to allow for simultaneous time-resolved measurement of device current/resistance. A temporary increase in current is noticed lasting for around 100 ns (which we attribute to a photothermal conductive effect^25^), after which the current drops to a lower level than pre-excitation, indicating a RESET (re-amorphization) process has occurred. The re-amorphization however is again partial, which is to be expected since the size of this exemplar device (1 × 1 µm^2^) is too large to be completely covered by our focused laser spot (which has a FWHM ≈ 0.5 µm). It is precisely the form of optically-induced switching demonstrated by Fig. 2(c) that could find application in optically-gated switches, though we note here that, due to the photothermal effects, the response (total switching) time is around 100 ns.

### Thermo-electrically switched devices

**Figure 3:**
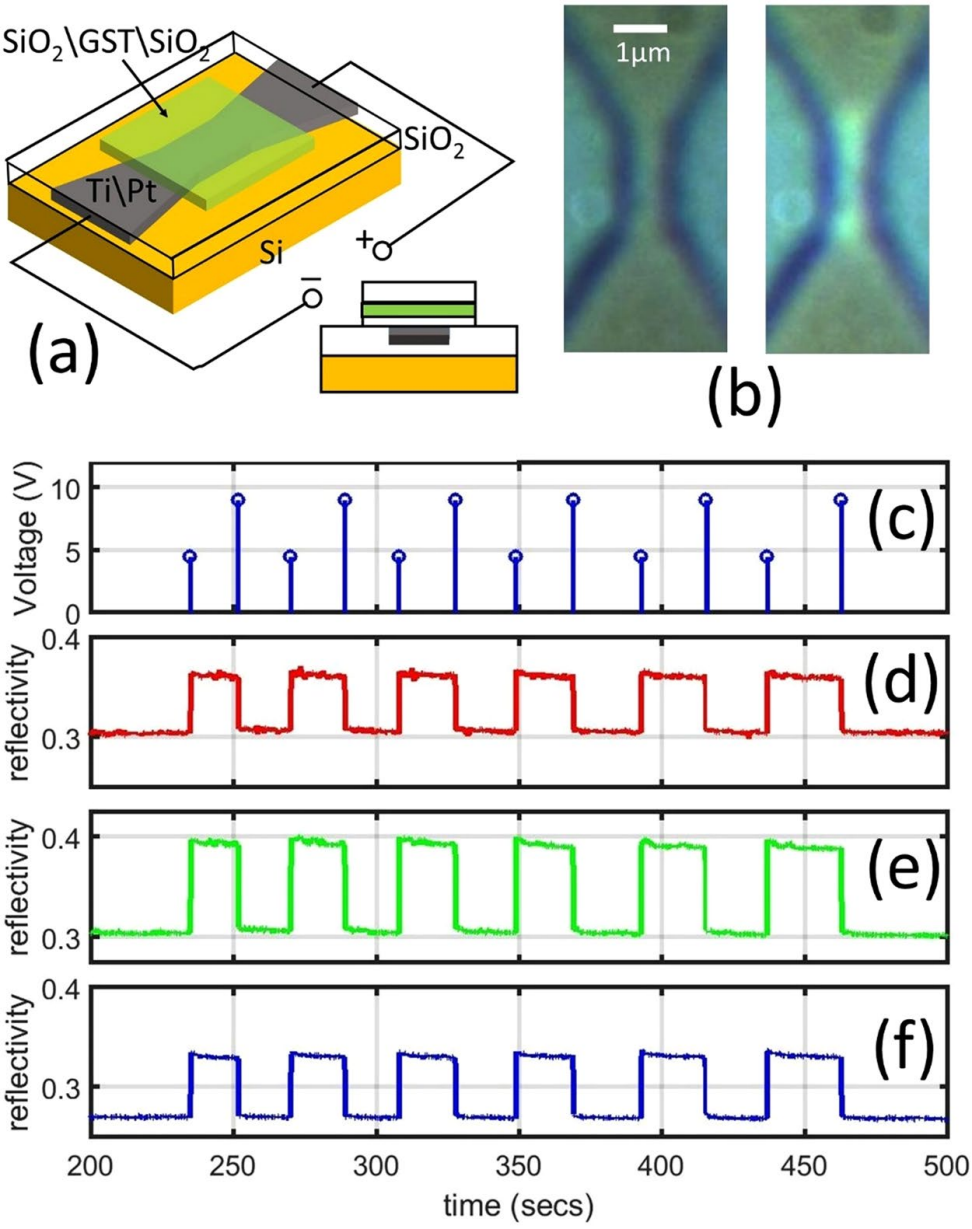
Thermally switched pixel-like devices. (**a**) Illustration of the thermally switched display pixel prototype: A Ti(5 nm)\Pt(45 nm) microheater electrode is patterned to provide a constriction in the centre and a GST(8 nm) layer sandwiched between two SiO_2_ layers (top/bottom thicknesses 50 nm and 5 nm respectively). (**b**) *In-situ* optical microscope image of the device (left) as-deposited (amorphous state) and (right) after a SET (crystallization) process. (**c**) Electrical excitations applied to the Pt microheater and (**d**) to (**f**) resulting real time reflectance changes for the red, green and blue channels respectively of the CMOS camera.

We now turn our attention to optically-active, thermo-electrically switched devices, by which we mean devices that can be optically probed (read) and in which the GST region is switched between RESET and SET states by thermal coupling to some form of heater (so no current flows through the GST region itself). One such a device is shown in Fig. 3(a) and (b) and comprises a microscale platinum heater (on a Si/SiO_2_ substrate) with a patterned GST layer on top, with the GST layer being sandwiched by two optically transparent SiO_2_ layers. Electrical pulses (Fig. 3(c)) are applied across the platinum heater electrodes, leading to Joule heating, with the heating greatest in the narrow (1 × 2 µm^2^) constricted heater region. By such means we were able to successfully cycle the GST region repeatedly between amorphous and crystalline states, as evidenced by the change in reflectance observed using *in-situ* white light microscopic imaging (Fig. 3(b)) and by reflectance measurements made via the RGB channels of our *in-situ* CMOS camera (see Figs 3(d) to (f)). We note that significant changes in reflectance occur at all three of the RGB wavelengths; specifically we observe reflectance changes of 20% (0.3 to 0.36) in the red, 30% (0.30 to 0.39) in the green and 22% (0.27 to 0.33) in the blue. It is such changes in the reflectance spectrum on phase-switching that lead to the potential use of phase-change devices for optoelectronic display applications (with the relative changes in reflectance in different parts of the spectrum being controlled in particular by the lower dielectric layer thickness – see ref. 4). Here we have shown that such changes can be induced by simple thermally-induced switching of the phase-change layer, and that cycling of relatively large areas of the phase-material can be achieved in such a manner (not usually the case using conventional ‘direct’ electrical switching). This demonstrates that thermally-switched devices are well-suited to the realization of large area phase-change pixels.

In summary, we have constructed a specialized probe station for determination of the optoelectronic, or mixed electrical-optical characteristics, of phase-change materials and devices. The probe station, while capable of “conventional” measurement such as measuring the electrical (optical) response of a device under electrical (optical) excitation, also provides new opportunities for exploring the interaction between electrical and optical behaviour and the development of new mixed-mode device functionalities. By way of examples we have explored the design, fabrication and operation of an optically-gated phase-change switch, as well as a thermally-switched microheater-based phase-change device potentially suited to the development of large-area pixels for phase-change display applications (and the emerging class of phase-change metadevices).

## Methods

### Cross-bar device fabrication

The cross-bar devices were fabricated using e-beam lithography, as described in more detail in Fig. S 1 of the Supplementary Information. Referring to Fig. S 1: (1) Electron beam lithography (EBL) is performed on a silicon substrate with a 50 nm thermal oxide layer on the top. (2) Reactive ion beam etching is used to create a trench 10 nm deep. (3) 5 nm of Ti followed by 5 nm of Pt is sputter deposited. (4) Lift-off of the PMMA leaves the Ti\Pt electrode embedded in the SiO_2_. (5) A second round of EBL is performed. (6) GST and ITO are then sputter deposited. (7) Lift off of the PMMA leaves a finished cross-bar structure.

(Microheater type devices were fabricated using a similar approach).

### Simultaneous optical-electrical excitation and measurement

Figure 1(a) displays the experimental setup. A pulsed or continuous wave (cw) laser with a wavelength of 405 nm is focused onto the sample surface in a diffraction limited spot using a 50x microscope objective with a N.A. of 0.50 (Olympus LMPLFLN50x). The objective has a long working distance equal to 10.6 mm, allowing the use of a pair of picoprobes (GGB Industries, 40A-GSG-200-EDP) for electrical measurements and so enabling high bandwidth, simultaneous electrical and optical access to the device under test. For further details of the objective lens arrangement, the picoprobes and the associated design of sample electrical contacts, refer to Figs. S 2 and S 3 in the Supplementary Information. The laser is a CNI (Changchun New Industries) laser diode with nominal maximum output power of 100 mW. The laser beam is collimated by a Thorlabs C671TME-405 aspheric lens (item 2 in Fig. 1a). The laser diode is mounted on a purposely made bias Tee (Avtech Electrosystems AVX-S1-P2-EX1). The diode is constantly biased at sub-threshold state by a constant current source (GWInstek PSS-3203). SET and RESET pulses are provided by two separate pulse generators (Tektronix AFG3101 and Avtech Electrosystems AVMR-2D-B) connected to the same bias Tee one at a time, controlled by a RF relay (Teledyne coax switch CCR-33S30-T). A computer controlled shutter (Thorlabs SHB05) is installed in the laser beam pathway. It is normally shut and opened only when the pulse generators are triggered. This avoids any undesired spurious laser irradiation reaching the sample device. The beam expander (item 3 in Fig. 1a) was removed during laser excitation of the sample (to increase the laser power available). The sample, together with the electrical probes (providing electrical connections to the device) are mounted on a piezoelectric precision stage (Physik Instrumente P-517.2CL, providing 100 μm range of movement in two lateral directions) sitting on the top of two manual stages (25 mm range of movement). This arrangement provides precise control of the position of the focused laser spot relative to the cross-bar device, as monitored by the CMOS camera. The microscope objective is mounted on a single axis piezo stage (Physik Instrumente, P-721) to provide precise light focusing. A 450 nm long pass filter is installed in front of the CMOS camera to protect it against damage when a cw laser is applied to the sample.

### Microheater type device measurements

The total resistance of the 1 × 2 µm^2^ Ti\Pt, microheater together with its contact pad is around 110 Ω. For electrical excitation of this device the 50 Ω shunt resistor (24) in Fig. 1(a) was replaced by a 70 Ω resistor to ensure a more effective electrical termination. The SET and RESET excitations were provided by a Tektronix AFG3101 pulse generator, with the SET pulse being of 50 ms duration and having rise/fall times of 50 ms, while the RESET pulse was of 50 ns duration and <1 ns rise/fall time. During excitation, an optical image of the microheater device under the illumination from a white light source (15 in Fig. 1(a)) is monitored by the CMOS camera (6 of Fig., 1(a), Thorlabs DCC1645C). The white light source is a LED flashlight (Night Searcher Tracker 240) that provides enough intensity stability to allow sensitive reflectivity measurements. The position of the device under test is detected by recognition software that provides feedback to the piezo stage (item 6 in Fig. S 2) for anti-drift purposes. The CMOS camera is colour sensitive, enabling us to differentiate reflectance response in the red, green and blue.

## Electronic supplementary material

Supplementary information
