# Supplementary material for: Phase-change devices for simultaneous optical-electrical applications
Source: Sci Rep. 2017 Aug 29;7:9688. doi: 10.1038/s41598-017-10425-8 (PMC5575275; doi:10.1038/s41598-017-10425-8)
Supplement: Supplementary file 1 — Supplementary information [file 41598_2017_10425_MOESM1_ESM.pdf]

Phase-change devices for simultaneous optical-electrical applications

Yat-Yin Au[1], Harish Bhaskaran[2] and C. David Wright[1]

[1]Department of Engineering, University of Exeter, Exeter, EX4 4QF, UK

[2]Department of Materials, University of Oxford, OX1 3PH, UK

Supplementary information

Device Fabrication: All devices in the main paper were fabricated using electron-beam lithography and reactive ion etching. The process steps in the fabrication of the cross-bar type device is shown below (steps for the microheater device were very similar).

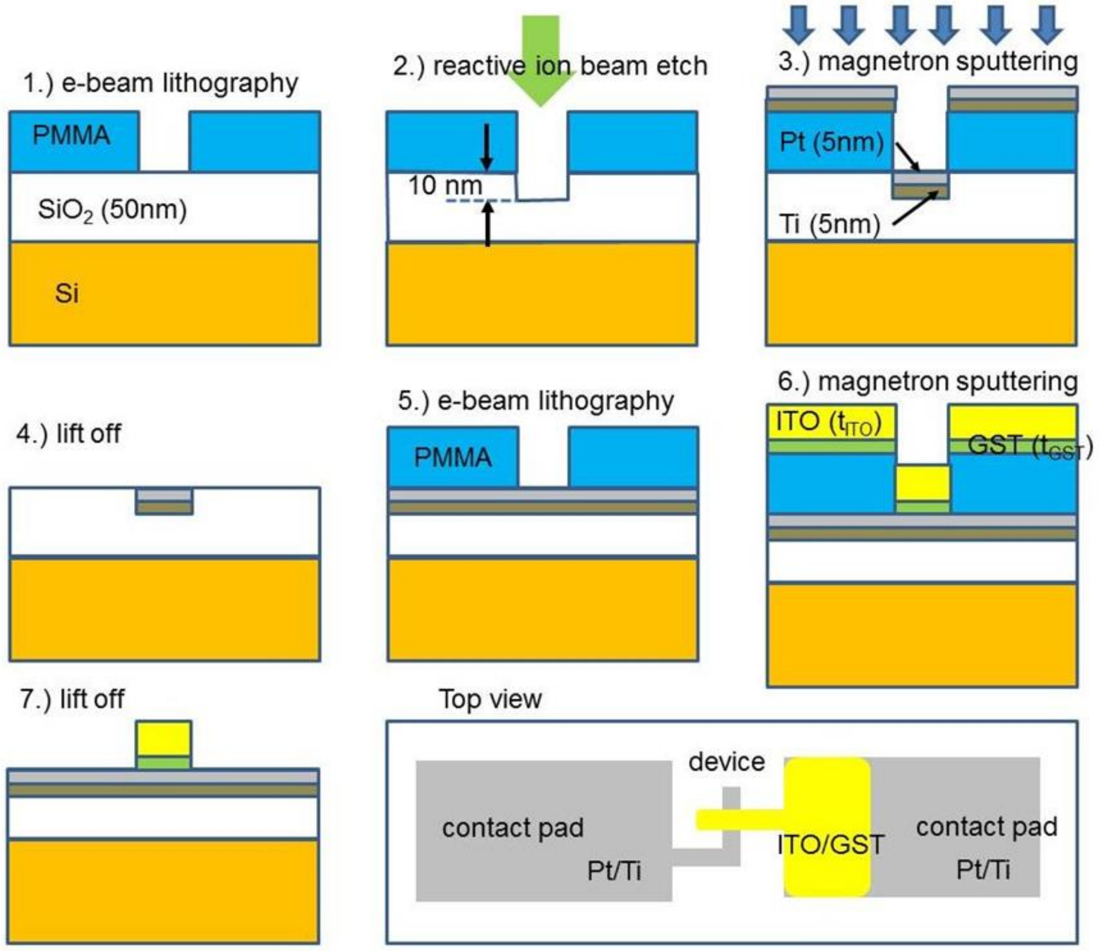

Figure S1. Illustration of the crossbar devices fabrication process. The reactive ion beam etch was done with Argon and CHF_3 flow at 15 sccm with plasma pressure at 45 mTorr, 50 W rf power for 30 secs. The Ti and Pt was deposited at a base vacuum of $2.6X10^{-7}$ Torr, with an Argon plasma $6.3X10^{-3}$ Torr with power equal to 30 W and 50 W respectively. The GST and ITO was deposited at a base vacuum of $2X10^{-6}$ Torr, with 25 W under an Argon plasma at $1X10^{-3}$ Torr.

Combined optical and electrical probing: Supplementary figures showing the detailed arrangement of the objective lens, electrical probes and the device GSG contacts that allow for simultaneous optical and electrical excitation and measurement are given below.

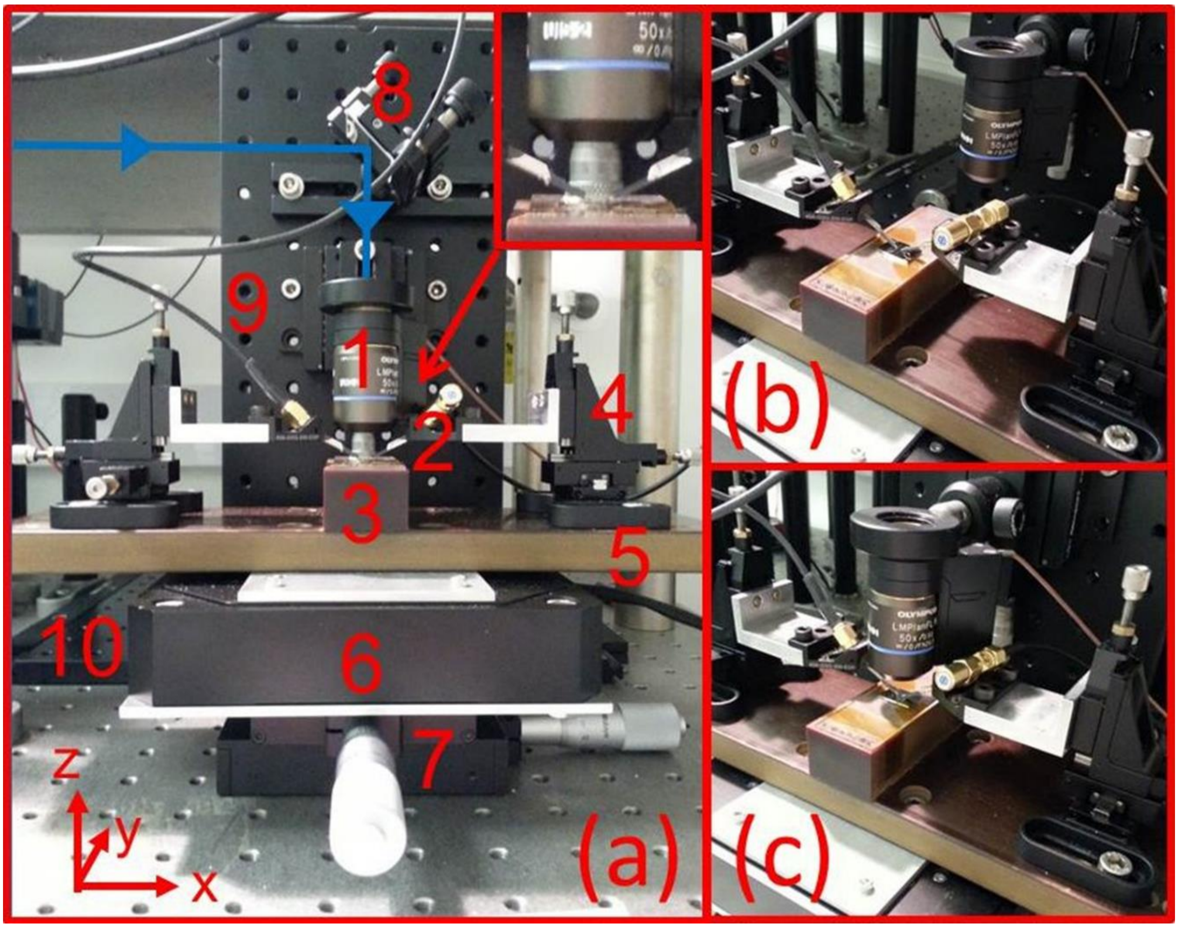

Figure S2. (a) Core component of the test platform: (1) Objective, (2) picoprobe, (3) sample, (4) manual micro-stage, (5) support stage, (6) lateral piezo stage, (7) lateral manual stage, (8) mirror, (9) breadboard and (10) horizontal sliding platform. Inset: Close up picture of the objective together with the picoprobes on a sample. (b) Objective pushed aside allows close inspection of picoprobe landing process. (c) Objective in measurement position.

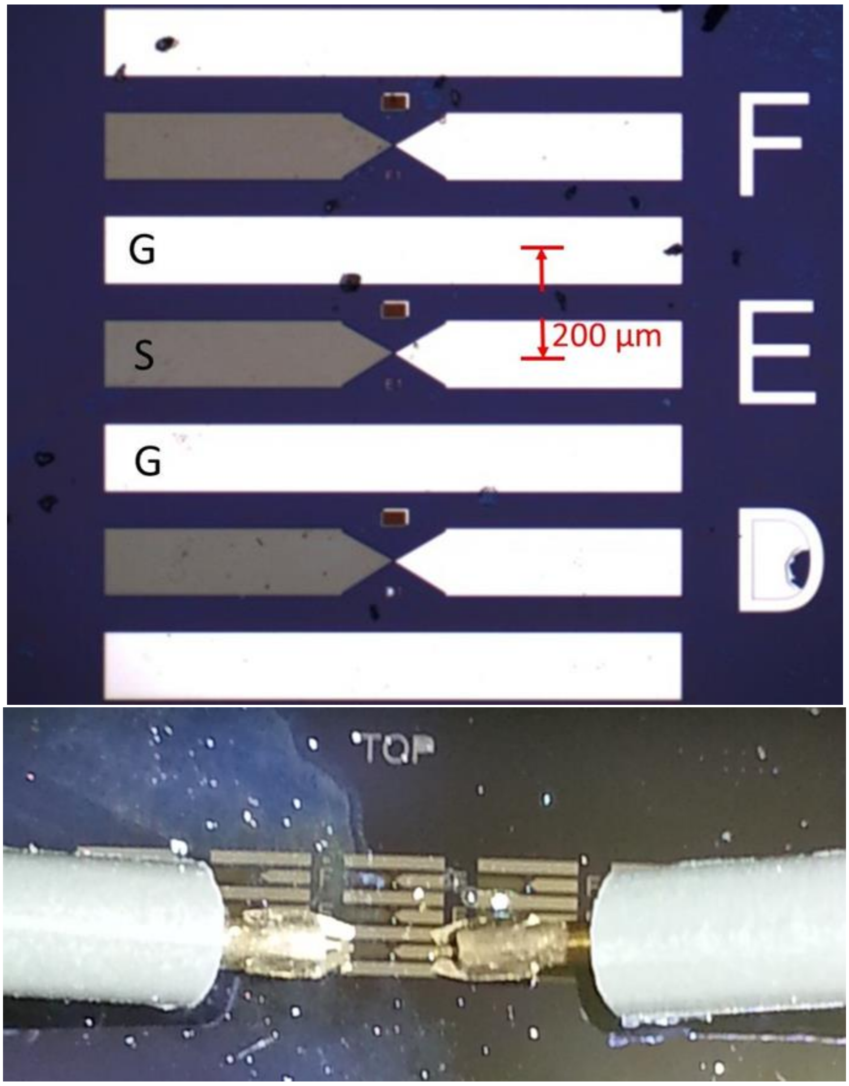

Figure S3. (Upper) Ground-Signal-Ground electrical contact pad design to provide fast electrical access to a phase change device located at the center. (Lower) Picoprobes in contact with the coplanar waveguide contact pad of a phase change device.
